# Do Differences in Cultivable Subgingival Species Exist between Different Periodontitis Stages and Grades?

**DOI:** 10.3290/j.ohpd.b875525

**Published:** 2021-01-26

**Authors:** Katja Tomšič, Katarina Rodič, Anja Sotošek, Petja Videmšek, Katja Seme, David Herrera, Mariano Sanz, Rok Gašperšič

**Affiliations:** a Student of Dentistry, Faculty of Medicine, University of Ljubljana, 1000 Ljubljana, Slovenia. Data acquisition, drafting of article.; b Student of Dentistry, Faculty of Medicine, University of Ljubljana, 1000 Ljubljana, Slovenia. Data acquisition, drafting of article.; c Student of Dentistry, Faculty of Medicine, University of Ljubljana, 1000 Ljubljana, Slovenia. Data acquisition, drafting of article.; d Student of Dentistry, Faculty of Medicine, University of Ljubljana, 1000 Ljubljana, Slovenia. Data acquisition, drafting of article.; e Professor, Institute of Microbiology and Immunology, Faculty of Medicine, University of Ljubljana, 1000 Ljubljana, Slovenia. Microbiological data analysis, drafting of article.; f Professor, ETEP (Etiology and Therapy of Periodontal Diseases) Research Group, Faculty of Dentistry, University Complutense of Madrid, 28040 Madrid, Spain. Conception and design of study, data analysis and interpretation, drafting and revising of article.; g Professor, ETEP (Etiology and Therapy of Periodontal Diseases) Research Group, Faculty of Dentistry, University Complutense of Madrid, 28040 Madrid, Spain. Conception and design of study, data analysis and interpretation, drafting and revising of article.; h Assistant Professor, Department of Oral Medicine and Periodontology, Faculty of Medicine, University of Ljubljana, 1000 Ljubljana, Slovenia. Conception and design of study, data analysis and interpretation, drafting and revising of article.

**Keywords:** periodontitis, stage, grade, Porphyromonas gingivalis, Aggregatibacter actinomycetemcomitans, Fusobacterium nucleatum

## Abstract

**Purpose:**

To investigate the subgingival microbiological profiles of patients with periodontitis, to determine their stage and grade scores and to evaluate the differences in the microbiota among different stages and grades.

**Materials and Methods:**

Sixty-seven (n = 67) periodontitis patients were selected. Periodontitis staging and grading, following the 2018 classification system, were defined. Following a clinical examination, subgingival samples were taken from the deepest periodontal pocket of each quadrant for cultivation, identification and quantification. The prevalence, proportion and counts of nine selected periodontal pathogens were determined, and differences between periodontitis stages III and IV and grades B and C were assessed.

**Results:**

All nine cultivable periodontal bacteria were detected, of which the most prevalent was *P. intermedia* (91.0%) and the least prevalent were *E. corrodens* (9.0%) and *C. ochracea* (9.0%). The frequency of detection of the two main target pathogens, *A. actinomycetemcomitans* and *P. gingivalis*, was 41.8% and 76.1%, respectively. The prevalence (grade B: 80.6%, grade C: 55.6%, p = 0.035) and total counts (grade B: 19.8 colony forming units – CFU/ml^-4^ (1.9–52.8); grade C: 4.0 CFU/ml^-4^ (0.0–26.4); p = 0.022) of *F. nucleatum* were statistically significantly higher in grade B than in grade C periodontitis patients, whereas the counts of *P. gingivalis* and *A. actinomycetemcomitans* were similar between grades and stages.

**Conclusion:**

Our study suggests that relevant differences between the various grades of periodontitis exist only in the numbers of *F. nucleatum*. Prevalence and quantities of other cultivable species between different stages and grades of periodontitis seem to be similar.

More than 700 different bacterial species have been identified in subgingival biofilms.^4^ Periodontitis-associated microbiota has been historically systemised into commensal microorganisms, principal periodontal pathogens and putative periodontal pathogens, according to criteria proposed by Socransky.^33^ Hence, three main periodontal pathogens with a strong association with periodontitis (*Porphyromonas gingivalis, Aggregatibacter actinomycetemcomitans* and *Tannerella forsythia*)^27,34,15^ and several putative periodontal pathogens (*Eikenella corrodens, Campylobacter rectus, enteric bacilli, Eubacterium nodatum, Fusobacterium nucleatum, Prevotella intermedia, Parvimonas micra, Streptococcus intermedius, Pseudomonas spp., Selenomonas spp., Staphylococcus spp*. and *Treponema denticola*)^37^ were identified on the basis of cross-sectional association studies, demonstration of bacterial virulence and immunologic responses in patients harbouring the selected microorganisms. Genetic analyses of periodontitis-associated bacteria that have demonstrated unanticipated genetic diversity within species, including genes of potential virulence factors, have changed the perception of bacterial species as a population of clones with variable pathogenic potential.^1,5,24^ However, according to Casadevall and Pirofski,^8^ the term “pathogen” should be viewed as obsolete, since the capacity to cause disease, even in strains with an impressive array of virulence factors, equally depends on host-dependent immunological responses and environmental risk factors. Hajishengallis, Darveau and Curtis^11^ proposed the keystone-pathogen hypothesis, defining a keystone pathogen as a low-abundance microorganism with high pathogenic potential that can cause an immune reaction in the host by remodelling a normally benign microbiota into a dysbiotic one. In the case of periodontitis, virulent strains of *P. gingivalis,* a minor constituent of plaque biofilms, possess the capacity to weaken the host’s inflammatory response and consequently change the growth and development of the entire biofilm; the equilibrium between the host and microbiota, therefore, collapses, resulting in periodontal tissue destruction.^11^

Carriage of putative periodontal pathogens may statistically significantly vary between different groups of periodontitis patients.^28^ However, the comparison between different groups of patients is hampered by the lack of a standardised epidemiological definition of periodontitis, as well as the fact that the same bacterial species have frequently been found in the dental biofilm of patients with healthy, as well as diseased periodontal tissues.^16^ Considering the range of identification methods, each with inherent advantages and disadvantages, that have been used to study the presence or absence of putative periodontal pathogens^30,9^ a definite conclusion regarding the prevalence of putative periodontal pathogens is even more difficult.

In the past, different forms of periodontal disease were observed and many of their classification systems questioned. Namely, whether different forms of periodontitis counted as individual diseases or just variations of a single disease. In addition, the previous classification system^3^ distinguished between chronic and aggressive periodontitis, which was not reflected in microbiological profiles of subgingival plaque samples, since comparative studies were not able to discriminate one form from another based on *A. actinomycetemcomitans* and *P. gingivalis* detection,^20,23^ an attribute originally defined as a secondary feature of aggressive periodontitis.^3^ In order to improve the identification, treatment and prevention of periodontitis, a new classification system has recently been proposed,^35^ whereby ‘chronic’ and ‘aggressive’ forms of periodontitis now belong to the same category: ‘periodontitis’. The disease is now categorised by stage and grade scores, which give a multidimensional view of the disease.^25^

Stage and grade scores are given according to several parameters. Staging is based on disease presentation in terms of severity and complexity, allowing for improved patient management and treatment planning, and encompasses four different levels: from stage I, which correlates with initial attachment loss and a mild clinical picture, to stage IV, which refers to severe breakdown of periodontal tissues in addition to occlusal collapse. Grading is based on evidence of progression and risk factors: it indicates patients’ susceptibility to disease and rate of progression. The primary criteria, defined by radiographic longitudinal data, bone loss versus age and biofilm deposits versus destruction, are modified by risk factors (smoking, diabetes) in order to determine patients’ appropriate grades, A, B or C. Grade A periodontitis is characterised by a slow rate of periodontitis progression, whereas patients with grade C have a rapid rate of progression.^35^ Important parameters considered by the classification are clinical characteristics, yet microbiologic profiles or microbiologic differences between individual stages and grades have not been determined.

Therefore, the aims of this investigation were to evaluate the prevalence of individual pathogens, their quantities and proportions in patients with periodontitis, and evaluate differences in microbiologic profiles between patients pertaining to different grade and stage categories. Our hypothesis was that differences exists in the prevalence and quantity of principal periodontal pathogens between different stage and grade categories.

## Materials and Methods

### Study Population

Individuals included in this cross-sectional study were patients consecutively seeking periodontal treatment at the Department of Oral Medicine and Periodontology, University Dental Clinic of Ljubljana, Slovenia. After evaluation of 1,152 patients, 67 (n = 67) systemically healthy individuals with untreated periodontitis were carefully selected according to the following criteria: age between 18 and 70 years; presence of at least 20 teeth; presence of at least one probing site in each jaw quadrant with a probing depth (PD) of at least 5 mm; and clinical attachment loss (CAL) of at least 3 mm. Patients who were pregnant or lactating, were medically compromised, had been treated for periodontitis in the past or had been treated with systemic antibiotics in the past 4 weeks, were excluded from the study. Excluded were also all patients with multiunit fixed partial dentures, removable dentures, implants and patients in need of complex rehabilitations due to masticatory disfunction, secondary occlusal trauma, extreme bite collapse, or presence of less than 20 remaining teeth. Smoking was not among exclusion criteria. All individuals signed an informed consent form. Study protocol was approved by the National Medical Ethic Committee (46/08/15).

### Clinical Examination and Microbiological Sampling

Each individual was given a full mouth clinical examination. The following parameters were assessed at six sites on each tooth: presence of plaque deposits using a dichotomous plaque index (PlI), PD, gingival recession (REC) and presence/absence of bleeding on probing (BOP). All measurements were performed by the same experienced and calibrated examiner using a manual Williams probe (POW6, Hu-Friedy, Chicago, IL, USA). CAL was calculated as a sum of PD and REC.

Protocol adjustments were made with the reference laboratory at the Faculty of Dentistry, University Complutense of Madrid, Spain, resulting in substantial agreement in detection of nine cultivable species.^14^ Based on the clinical examination of each individual, four sites were chosen for microbiological sampling – one site with the deepest PD per jaw quadrant. Immediately after clinical examination, the sites were first cleansed of plaque and then dried using cotton rolls and gentle air drying. Next, two absorbent paper points (0.30 mm in diameter, Maillefer, Ballaigues, Switzerland) in a row were inserted into the chosen periodontal pocket and removed after 10 s. Paper points were placed in sample tubes containing 1.5 ml of reduced transport fluid (RTF) and kept at room temperature. They were transported to the Institute of Microbiology, Faculty of Medicine, University of Ljubljana, Slovenia, within 4 h for further processing.

Samples were serially diluted using phosphate buffer saline (PBS). 100 ml aliquots were then plated onto a blood agar medium (Oxoid No. 2; Oxoid, Basingstoke, UK) with 5% horse blood, haemin (5 mg/L) and menadione (1 mg/L). After 7–14 days of anaerobic incubation (80 % N_2_, 10% H_2_, 10% CO_2_ at 37°C) blood agar plates were examined and bacterial colonies were identified. The number of colonies of *Eikenella corrodens, Parvimonas micra, Fusobacterium nucleatum, Prevotella intermedia, Campylobacter rectus* and *Capnocytophaga ochracea* were determined after 7 days, while the number of colonies of *Tannerella forsythia* and of *Porphyromonas gingivalis* were determined after 14 days.

The diluted samples were also plated onto Dentaid-1 medium^36^ and incubated for 3 days in air with 5% CO_2_ at 37°C in order to isolate, grow and determine the number of *Aggregatibacter actinomycetemcomitans* colonies after 3–5 days, as well as onto MacConckey agar in order to determine the presence of enteric bacteria after 1–2 days of aerobic incubation at 37°C.

Bacterial colonies were identified based on colony morphologies of specific bacteria using a ring-light-equipped stereomicroscope Olympus SZX7 (Olympus, Tokyo, Japan), Gram staining, aerotolerance, catalase production and matrix-assisted laser desorption/ionisation time-of-flight mass spectrometry (MALDI-TOF MS) (MBT COMPASS 4.1, Microflex, Bruker Daltonics, Bremen, Germany).

The number of colonies for each bacterial species were determined on plates with a total colony count of 30–300 colonies; the numbers of colonies in 1 ml of sample (colony forming units, CFU/ml) were then calculated. Total numbers of colonies on each plate were also counted so that proportions of individual colonies based on the total number of anaerobic bacteria could be calculated.

### Periodontitis Stage Score Determination

Severity was determined based on (see Supplemental Table 1): interdental CAL at site of greatest loss (distal PD on last molars were not included), radiographic bone loss (RBL) in percentage and tooth loss. Complexity was determined based on (see Supplemental Table 2): intrabony defects and/or furcation involvement class II or III, PD 4–5 mm or ≥6 mm. When establishing the stage score (see Supplemental Table 3), complexity was considered as a modifier to severity.

### Periodontitis Grade Score Determination

Grade was determined based on (see Supplemental Table 4): the quotient of RBL in percentage and patients’ age and the proportion of plaque-covered probing surfaces as a quotient of probing surfaces with PDs of ≥5 mm and tooth loss (indicator of destruction). When establishing the grade score (see Supplemental Table 5), modifying factors (smoking) were considered.

### Statistical Analysis

Categorical variables were shown as frequencies and percentages. Non-normally distributed continuous variables were shown as medians with interquartile ranges. The association between the presence of each periodontal pathogen and stage or grade score was tested by multiple logistic regression. Each periodontal pathogen was included as an independent variable with age and gender as cofounders and stage or grade scores as independent variables. The odds ratios (OR) of having a higher stage or grade score for the presence of each periodontal pathogen was calculated with 95% confidence intervals (CI), adjusted for age and gender.

In order to test whether there was a difference in the total counts of each periodontal pathogen, according to stage or grade score, an analysis of covariance was applied with periodontal pathogen type as a repeated measures factor, gender and stage or grade as a between subject factor, age as a covariate and periodontal pathogen total counts as a dependent variable. Since the distribution of periodontal pathogen counts was highly positively skewed, the natural logarithm of pathogen counts increased by one (as logarithm can be calculated for positive values) was calculated prior to the analysis. There were no adjustments for multiple comparisons. The level of statistical significance was set to α = 0.05.

## Results

### Cross-Sectional Clinical and Microbiological Results

The average age of all 67 patients was 44.4 (standard deviation – SD 10.8) years. More than half were men (56.7%). The majority were non-smokers (74.6%), while the numbers of smokers and ex-smokers were the same (13.4%). On average, patients had 25 (SD 2.2) teeth. The average number of pockets with PD of >4 mm was 44.0 (SD 27.4). The average PD was 3.9 (SD 0.5) mm and the average CAL was 4.5 (SD 0.8) mm. The average PlI was 34.4% (SD 21.9) and the average proportion of sites with BOP was 41.1% (SD 38.2).

Most patients were allocated to stage III (48%) or stage IV (45%), and grade B (54%) or grade C (40%). The numbers and proportions of patients allocated to each stage and grade are shown in Table 1.

**Table 1:** Allocation of periodontitis patients based on their stage and grade of periodontitis

GRADE					
		A	B	C	Total
STAGE	I	0 (0%)	0 (0%)	0 (0%)	0 (0%)
	II	1 (1%)	4 (6%)	0 (0%)	5 (7%)
	III	2 (3%)	20 (30%)	10 (15%)	32 (48%)
	IV	1 (1%)	12 (18%)	17 (25%)	30 (45%)
	Total	4 (6%)	36 (54%)	27 (40%)	67 (100%)

All nine observed periodontopathogens were found in the analysed plaque samples. *A. actinomycetemcomitans* was found in 28 patients (41.8%), *P. gingivalis* in 51 patients (76.1%), *P. intermedia* in 61 patients (91.0%), *T. forsythia* in 57 patients (85.0%), *P. micra* in 60 patients (90.0%), *F. nucleatum* in 47 patients (70.1%), *C. rectus* in 31 patients (46.3%), *E. corrodens* in 6 patients (9.0%) and *C. ochracea* in 6 patients (9.0%). No enteric rods were detected.

In the plaque samples analysed, the highest and lowest proportion and median total counts belonged to *P. gingivalis* and *A. actinomycetemcomitans*, respectively. The proportions and median total counts of other periodontopathogens were low to moderate (Table 2).

**Table 2:** Proportions (in percentage) and total counts (in log of colony forming units, CFU/ml) of periodontal pathogens in plaque samples of patients with periodontitis. The values represent median scores with interquartile range (IQR) for 67 patients

Bacterial species	Proportion (range)	Total counts (range)
*A. actinomycetemcomitans*	0.0 (0.0–0.6)	4.2 (3.8–4.6)
*P. gingivalis*	6.8 (0.3–20.8)	6.4 (5.8–6.8)
*P. intermedia*	2.9 (0.9–8.6)	5.8 (5.3–6.2)
*E. corrodens*	0.0 (0.0–0.0)	5.6 (5.0–6.0)
*F. nucleatum*	0.9 (0.0–2.5)	5.4 (5.0–5.8)
*P. micra*	3.8 (1.5–6.0)	5.8 (5.1–6.2)
*C. rectus*	0.0 (0.0–1.8)	5.4 (5.1–6.2)
*C. ochracea*	0.0 (0.0–0.0)	5.0 (4.8–5.1)
*T. forsythia*	3.9 (1.7–8.2)	5.8 (5.5–6.2)

*CFU/ml – colony forming units in 1 ml of sample.

### Microbiological Differences According to Stage and Grade Scores

The logistic regression model showed no association between the presence of each periodontal pathogen and stage score, when controlling for age and gender of patients (Table 3). As for grade (Table 4), an association was found between the presence of *F. nucleatum* and grade score (p = 0.035), when controlling for age and gender of patients. A higher share of patients with grade B periodontitis (80.6%) were positive for *F. nucleatum* in comparison to patients with grade C (55.6%) periodontitis. The odds of grade C periodontitis patients having *F. nucleatum* were 0.3 (95% CI: 0.1 – 0.92) times lower in comparison to patients with grade B. No other pathogens were associated with grade scores.

**Table 3:** Association between the presence of periodontal pathogens and stage score (results of logistic regression). Data shown as frequencies (percentages)

	Stage III (n = 32)	Stage IV (n = 30)	aOR (95 % CI)	P
*A. actinomycetemcomitans*	14 (43.8)	13 (43.3)	0.93 (0.33; 2.62)	0.885
*P. gingivalis*	24 (75.0)	24 (80.0)	1.43 (0.39; 5.17)	0.588
*P. intermedia*	30 (93.8)	28 (93.3)	0.93 (0.11; 8.01)	0.948
*T. forsythia*	27 (84.4)	26 (86.7)	1.17 (0.27; 5.09)	0.832
*P. micra*	27 (84.4)	29 (96.7)	7.85 (0.78; 79.42)	0.081
*F. nucleatum*	19 (59.4)	23 (76.7)	2.25 (0.72; 7.04)	0.163
*C. rectus*	12 (37.5)	16 (53.3)	2.34 (0.79; 6.91)	0.123
*E. corrodens*	1 (3.1)	4 (13.3)	5.14 (0.48; 55.56)	0.177
*C. ochracea*	2 (6.3)	4 (13.3)	2.08 (0.33; 13.15)	0.437

^a^OR = Odds ratios adjusted for age and gender; CI, confidence interval.

**Table 4:** Association between the presence of periodontal pathogens and grade score (results of logistic regression). Data shown as frequencies (percentages)

	Grade B (n = 36)	Grade C (n = 27)	aOR (95 % CI)	P
*A. actinomycetemcomitans*	15 (41.7)	11 (40.7)	0.97 (0.35; 2.69)	0.949
*P. gingivalis*	27 (75.0)	21 (77.8)	1.15 (0.34; 3.86)	0.819
*P. intermedia*	32 (88.9)	26 (96.3)	3.89 (0.37; 40.50)	0.255
*T. forsythia*	32 (88.9)	22 (81.5)	0.50 (0.12; 2.1)	0.340
*P. micra*	32 (88.9)	24 (88.9)	1.05 (0.21; 5.23)	0.957
*F. nucleatum*	29 (80.6)	15 (55.6)	0.30 (0.10; 0.92)	0.035*
*C. rectus*	16 (44.4)	14 (51.9)	1.48 (0.53; 4.14)	0.454
*E. corrodens*	3 (8.3)	3 (11.1)	1.24 (0.22; 7.04)	0.805
*C. ochracea*	3 (8.3)	3 (11.1)	1.24 (0.22; 6.85)	0.806

^a^OR = Odds ratios adjusted for age and gender; CI, confidence interval; * = statistical significance.

The total counts of periodontal pathogens, divided according to their stage and grade scores, are shown in Table 5. Using age as a covariate, grade, gender and smoking as intermeasure factors, the natural logarithm of total counts as a dependent variable and periodontal pathogens as repeated measure factors, the analysis of covariance showed a statistically significant interaction between periodontal pathogens and grade scores (p = 0.041). Post-hoc tests with the Sidak correction for multiple comparisons showed a statistically significant difference in the total count of *F. nucleatum* between igrade B and grade C (p = 0.022) periodontitis patients. Patients with grade B periodontitis had higher total counts of *F. nucleatum* in comparison to patients with grade C periodontitis.

**Table 5:** Total counts (colony forming units, CFU/ml^-4^) of periodontal pathogens in plaque samples of patients with periodontitis, classified according to their stage and grade scores. Data shown as medians (interquartile range, IQR)

	Stage III	Stage IV	Grade B	Grade C
*A. actinomycetemcomitans*	0.0 (0.0; 1.0)	0.0 (0.0; 1.73)	0.0 (0.0; 0.7)	0.0 (0.0; 1.4)
*P. gingivalis*	43.6 (3.3; 238.0)	204.5 (79.2; 564.9)	69.3 (3.3; 432.8)	165.0 (29.0; 422.4)
*P. intermedia*	57.4 (9.9; 145.2)	79.9 (13.2; 145.2)	75.9 (16.9; 208.0)	33.0 (12.5; 131.8)
*T. forsythia*	46.2 (6.9; 92.4)	67.0 (33.0; 217.8)	59.4 (22.1; 151.9)	66.0 (26.4; 138.6)
*P. micra*	33.0 (5.6; 125.5)	49.2 (26.3; 165.0)	62.7 (7.6; 161.5)	33.0 (8.5; 112.2)
*F. nucleatum*	9.9 (0.0; 39.6)	16.4 (1.3; 46.2)	19.8 (1.9; 52.8)	4.0 (0.0; 26.4)
*C. rectus*	0.0 (0.0; 12.2)	6.6 (0.0; 46.2)	0.0 (0.0; 22.5)	3.3 (0.0; 26.4)
*E. corrodens*	0.0 (0.0; 0.0)	0.0 (0.0; 0.0)	0.0 (0.0; 0.0)	0.0 (0.0; 0.0)
*C. ochracea*	0.0 (0.0; 0.0)	0.0 (0.0; 0.0)	0.0 (0.0; 0.0)	0.0 (0.0; 0.0)

When stage, instead of grade, was included in the ANCOVA model as an intermeasure factor, no statistically significant interaction between periodontal pathogen type and grade score could be found (p = 0.642).

## Discussion

All nine cultivable periodontopathogens were detected in dental plaque samples of Slovene periodontitis patients. The highest proportion was found for *P. gingivalis* and the lowest for *A. actinomycetemcomitans*. Higher prevalence and total counts of *F. nucleatum* were found in grade B rather than grade C periodontitis patients. Interestingly, no statistically significant differences between stages and grades were found for *A. actinomycetemcomitans* and *P. gingivalis*.

Cultivation-based techniques are still considered a gold standard of identification, quantification, tracking of changes in microbiologic profiles during treatments and establishing bacterial sensitivities to antibiotics.^30,36^ Before the evaluation of our patients, protocol adjustments were made with the reference laboratory at the Faculty of Dentistry, University Complutense of Madrid, Spain.^14^ Since similar protocol adjustments with reference laboratories had been carried out in the Netherlands, Colombia and Chile,^13,29^ comparison with these laboratories is also possible. The only difference is that we identified bacterial species by means of MALDI-TOF mass spectrometry, as opposed to biochemical tests used in the other laboratories. MALDI-TOF mass spectrometry has become a popular method for microbial identification in clinical microbiology laboratories around the world, providing a fast, cheap and reliable tool for the identification of bacteria and fungi cultivated on agar plates or in liquid media, based on automated analyses of the mass distribution of bacterial proteins.^26,31^

All 9 usually tested cultivable bacteria were identified in the samples of Slovene patients. The prevalence of *A. actinomycetemcomitans*, *P. intermedia*, *T. forsythia*, *P. gingivalis* and *P. micra* is high in Slovene patients, while the proportions of these bacteria in Slovene patients are moderate when compared to the other populations. It is also important to note that enterococci were not found in Slovene patients. Due to the calibration between laboratories, the diversity in detection frequencies (prevalence) and proportions of bacteria between the above described populations cannot be attributed to methodological differences between individual laboratories. They could be explained by genetic factors,^17^ diverse oral hygiene habits, the level of dental healthcare, exposure to tobacco products, the (over)use of antibiotics and the differences in host responses.^18^

For this research, it was necessary to determine stage and grade scores of patients previously categorised under ‘chronic periodontitis’, as proposed by the new classification of periodontitis.^35^ The new classification criteria were modified resulting in the following limitations. With regards to stage, the causes of tooth loss often could not be determined since patients themselves did not know or remember the reasons for extraction. Therefore, all tooth loss was attributed primarily to periodontitis in order to avoid subjective guesses of the causes. Furthermore, in order to keep the investigation objective, final severity and complexity scores were calculated as averages of the separate criteria under investigation. As for the determination of complexity, only two criteria were considered, since the inclusion criteria excluded patients in need of complex rehabilitations due to masticatory disfunction, secondary occlusal trauma, extreme bite collapse or presence of less than 20 remaining teeth. The final stage score was calculated as the average of the severity and complexity scores; whenever the average score was in the arithmetic middle, the severity score was taken as the stage score (example: if severity = 3 and complexity = 2, the average score is 2.5; stage is therefore 3) since the initial stage should be determined using CAL, a category of severity.^35^ When determining the grade score, the two main dimensions, the primary criteria and grade modifiers, were taken into account. Due to the cross-sectional design of our investigation, direct evidence (longitudinal data) was not available, so only indirect evidence of progression was considered. The case phenotype, which assesses the relationship between biofilm deposits and destruction, was objectified for the purpose of a more uniform system of grade score determination across patients. As for the risk factors, the main dilemma was how to classify ex-smokers, since this was not defined in the new classification system. All included ex-smokers had previously smoked for at least 15 consecutive years; most, more than 10 cigarettes a day. Therefore, ex-smokers that had stopped smoking more than a year ago were allocated to grade A and ex-smokers that had stopped less than a year ago were allocated to grade B. The primary criteria score was calculated as the average primary criteria score of all three separate criteria, while the final grade score was calculated as the average of the primary criteria and grade modifier scores; whenever the average score was in the middle of two scores, the grade modifier score was taken as the grade score since a risk factor should shift the grade score to a higher value independently of the primary criterion.^35^

The last aim of this investigation was to evaluate the differences in prevalence and total counts (log CFU/ml) of the examined periodontal pathogens between patients of each grade and stage category. Statistical comparisons of microbiological profiles were performed between stage III and stage IV patients, as well as between grade B and grade C patients, while stage II and grade A patients were not taken into consideration due to the small number of patients allocated to these groups (stage II: 5 patients, grade A: 4 patients). We found low OR of grade C periodontitis patients harbouring *F. nucleatum* within their subgingival plaque when compared to grade C periodontitis patients. *F. nucleatum*, an orange complex putative pathogen, has been found to be the main cause of initial periodontal destruction; its total counts characteristically increase as gingivitis develops into periodontitis, thus adding to the destruction of periodontal tissue.^21^ In addition, antibody titres to *F. nucleatum* are higher in periodontitis than gingivitis patients.^12^ It is usually found in combination with other periodontal pathogens such as *P. gingivalis*^10^ and *T. forsythia*.^32^ Even though the bacteria itself possesses several virulence factors, its cooperative manner of destruction shows synergistic pathogenicity in combined infections. Being a bridge bacterium, which connects primary colonisers, offers structural support to dental biofilms and shapes host responses,^7^ its role, according to our results, seems to be more important in forms of periodontitis with moderate (grade B periodontitis) than higher (grade C periodontitis) rates of progression. On one hand, the presence of *F. nucleatum* results in the destruction of periodontal tissue, while on the other hand, it may prevent an overly aggressive course of disease progression. In addition, the presence of *F. nucleatum* has been documented in both healthy and diseased periodontal sites, suggesting the existence of several bacterial stains with varying degrees of pathogenic potential, relating to different levels of disease activity.^6^ This too may partially explain the relationship between *F. nucleatum* and grade B patients, rather than grade C patients.

Even though statistically significant differences in the prevalence and proportions of *A. actinomycetemcomitans* were expected between individual stage and grade scores, the statistical analyses showed no such results. As *A. actinomycetemcomitans* has often been identified in subgingival samples of healthy individuals without any clinical signs of periodontal disease, the existence of *A. actinomycetemcomitans* strains lacking important virulent factors could be considered as a possible explanation of our results.^20^ However, as a detailed evaluation of *A. actinomycetemcomitans* revealed that almost all *A. actinomycetemcomitans* strains possessed a plethora of the most important virulent factors, including leukotoxin,^23^ such an explanation seems less likely. Nevertheless, in two of our *A. actinomycetemcomitans* isolates, the deletion of nucleotides in the CdtB genome could be responsible for the inactivation of cytolethal distending toxin (Cdt), which can notably reduce the toxic potential of this strain.^23^ We may conclude that, similarly to *A. actinomycetemcomitans*, the lack of differences in detection frequencies and counts of other cultivable bacteria across the stages and grades of periodontitis could be partially explained by differences in virulence capacity of particular strains and by different individual host responses that ultimately determine the phenotype and biology of periodontitis.

## Conclusion

Cultivable principal and putative periodontal pathogens are frequently found among periodontitis patients from Slovenia. The prevalence of *P. gingivalis* and *A. actinomycetemcomitans* was 76% and 42%, respectively. According to the new classification system, statistically significant differences in the microbiological profiles between grade B and C periodontitis patients were found in the proportion and total counts of *F. nucleatum*; these were higher in grade B compared to grade C periodontitis patients. As for most principal periodontal pathogens, detection frequencies and counts did not differ between different stages and grades.

## Figures and Tables

**Supplemental Table 1:** Colour combinations for severity score determination

Interdental CAL at site of greatest loss (mm)	Radiographic bone loss (%)	Tooth loss	SEVERITY
≥5	15–33%	0	2
≥5	15–33%	≤4	2–3
≥5	15–33%	≥5	2–3
≥5	33–50%	0	2–3
≥5	33–50%	≤4	3
≥5	33–50%	≥5	3
≥8	33–50%	0	2–3
≥8	33–50%	≤4	3
≥8	33–50%	≥5	4
≥8	>50%	0	3–4
≥8	>50%	≤4	4
≥8	>50%	≥5	4

White: SEVERITY 2; Pink: SEVERITY 3; Red: SEVERITY 4; CAL, clinical attachment loss.

**Supplemental Table 2:** Colour combinations for complexity score determination

Furcation involvement class (II or III) or intrabony defects	Probing depth (mm)	COMPLEXITY
No	4–5	2
No	≥6	2
Yes	4–5	2–3
Yes	≥6	3

White: COMPLEXITY 2; Pink: COMPLEXITY 3.

**Supplemental Table 3:** Colour combinations for final stage score determination

SEVERTIY	COMPLEXITY	STAGE
2–3	2	II
2–3	3	III
3	2	III
3	2–3	III
3	3	III
3–4	2	III
3–4	3	III
4	2	III
4	2–3	III
4	3	IV

**Supplemental Table 4:** Colour combinations for primary criterion determination

RBL/age	% plaque/% probing depths ≥5	Tooth loss	PRIMARY CRITERION:
0.25–1	>1.2	0	A-B
0.25–1	1 ± 0.2	0	B
0.25–1	<0.8	0	B-C
0.25–1	>1.2	1–5	B
0.25–1	1 ± 0.2	1–5	B
0.25–1	<0.8	1–5	B-C
0.25–1	>1.2	≥6	B-C
0.25–1	1 ± 0.2	≥6	B-C
0.25–1	<0.8	≥6	C
>1	>1.2	0	B
>1	1 ± 0.2	0	B-C
>1	<0.8	0	C
>1	>1.2	1–5	B-C
>1	1 ± 0.2	1–5	C
>1	<0.8	1–5	C
>1	>1.2	≥6	C
>1	1 ± 0.2	≥6	C
>1	<0.8	≥6	C

White: GRADE A; Light blue: GRADE 2; Dark blue: GRADE C.

**Supplemental Table 5:** Colour combinations for final grade score determination

Primary criterion	Modifying factor (smoking)	GRADE
B	No, or ex-smoker (stopped >1 year ago)	B
B	Yes 1–10/day, or ex-smoker (stopped <1 year ago)	B
B	Yes more than 10/day	C
B-C	No, or ex-smoker (stopped >1 year ago)	B
B-C	Yes 1–10/day, or ex-smoker (stopped <1 year ago)	B
B-C	Yes more than 10/day	C
C	No, or ex-smoker (stopped >1 year ago)	C
C	Yes 1–10/day OR ex-smoker (stopped <1 year ago)	C
C	Yes more than 10/day	C

White: GRADE A; Light blue: GRADE 2; Dark blue: GRADE C.
